# Krebs von den Lungen 6 (KL-6) levels in COVID-19 ICU patients are associated with mortality

**DOI:** 10.1186/s44158-022-00064-5

**Published:** 2022-08-20

**Authors:** Giuliana Scarpati, Daniela Baldassarre, Massimo Boffardi, Vincenzo Calabrese, Edoardo De Robertis, Graziella Lacava, Filomena Oliva, Pasquale Pagliano, Gabriele Pascale, Giovanni L. Tripepi, Ornella Piazza

**Affiliations:** 1 Department of Medicine, Surgery and Dentistry “Scuola Medica Salernitana, ” University of Salerno, Baronissi, SA Italy; 2 Salerno University Hospital “San Giovanni di Dio e Ruggi D’Aragona’, Cava de’ Tirreni, SA Italy; 3 Department of Clinical and Experimental Medicine, University of Messina, Messina, Italy; 4 Department of Medicine and Surgery, University of Perugia, Perugia, Italy; 5 CNR IFC Reggio Calabria, Reggio Calabria, Italy

**Keywords:** COVID-19, KL-6, ARDS, MUC1, ICU

## Abstract

**Background:**

Krebs von den Lungen 6 (KL-6) is a high-molecular-weight mucin-like glycoprotein, which is also known as MUC1. KL-6 is mainly produced by type 2 pneumocytes and bronchial epithelial cells, and, therefore, elevated circulating KL-6 levels may denote disorders of the alveolar epithelial lining.

The objective of this study is to verify if KL-6 serum level might support ICU physicians in predicting mortality, risk stratifying and triaging severe COVID-19 patients.

**Methods:**

A retrospective cohort study, including all the COVID-19 patients who measured KL-6 serum values at least once during their ICU stay, was performed. The study sample, 122 patients, was divided in two groups, according to the median KL-6 value at ICU admission (median log-transformed KL-6 value: 6.73 U/ml; group A: KL-6 lower than the median and group B: KL-6 higher than the median).

**Results:**

One-hundred twenty-two ICU patients were included in this study. Mortality was higher in group B than in group A (80 versus 46%; *p* < 0.001); both linear and logistic multivariate analyses showed ratio of arterial partial pressure of oxygen to fraction of inspired oxygen (P/F) significantly and inversely related to KL-6 values.

**Conclusion:**

At ICU admission, KL-6 serum level was significantly higher in the most hypoxic COVID-19 patients and independently associated with ICU mortality.

## Background/rationale

COVID-19 mainly affects the respiratory system by causing a low oxygenation index, even if clinical manifestations can be not very prominent in many hypoxic patients, with no complaint of dyspnoea, no significant increase in respiratory rate, and no respiratory distress (as in the “silent hypoxia” COVID-19 clinical scenario) [1]. It has been reported that about 33% of COVID-19 victims developed acute respiratory distress syndrome (ARDS) [2]. COVID-19 ARDS (CARDS) patients should be admitted to the intensive care unit (ICU), the elderly and those with comorbidities being at highest risk of death [3, 4]. CARDS was recently classified into 3 different categories depending on hypoxemia [5]: mild hypoxemia (between 200 and 300 mmHg), mild-moderate (150 to 200 mmHg), and moderate-severe hypoxemia (< 150 mmHg). CARDS patients show some differentiations with ARDS caused by other factors [4], but type 2 pneumocytes damage or transformation is a very relevant element in both CARDS and ARDS by other viral causes. In fact, in case of alveolar type 2 cells injury, removal of alveolar oedema fluid is compromised. Moreover, type 2 cells harm reduces the production and turnover of surfactant, which is associated with poor outcome. Mortality of CARDS is evolving, thanks to the efforts of the international scientific community. Extracorporeal membrane oxygenation (ECMO) has been used to treat COVID-19 ARDS, even if there are still debates on ethical considerations and end-of-life decision-making [6]. Extracorporeal CO2 removal systems (ECCO2R), developed to correct hypercapnia and promoting ultra-protective ventilation protocols, have been applied to COVID patients too [7].

The Krebs von den Lungen 6 (KL-6) protein is a high-molecular-weight mucin-like glycoprotein, also known as MUC1 [5, 8], produced by type 2 pneumocytes and bronchial epithelial cells, which may provide insight into the ARDS pathophysiology. KL-6 is known as an immunological biomarker reflecting the severity and progression of interstitial lung disease (ILD), a form of chronic fibrosing interstitial pneumonia with various aetiology [9]. In a recent systematic review [10], KL-6 concentration was higher in severe COVID-19 patients than in non-severe patients (95% *CI*: 0.99–1.5; *p* < 0.001). However, in this systematic review, including 136 severe COVID-19 patients, the criteria for COVID-19 classifications as severe and non-severe were not homogeneous among the 6 covered studies.

### Objectives

Since elevated circulating levels of KL-6 indicate disruption of the alveolar epithelial lining, we hypothesized that it could be advantageous analysing KL-6/MUC1 in a group of severe COVID-19-positive patients, to better risk stratify and triage them. Central question of this study was investigating the association between KL-6 serum levels and ICU COVID-19 mortality.

## Methods

### Study design and setting

This is a single-centre, retrospective observational study performed at the Salerno University COVID Hospital “G. Da Procida”, which was designated as a COVID-only medical centre in Salerno, Italy, on the 8th of October 2020.

### Participants

The ICU admission was reserved to severe COVID-19 patients. According to the National Institute of Health (NIH) guidelines [11], critical patients are identified as follows:

#### Severe illness

Individuals who have *SpO2* < 94% on room air at sea level, a ratio of arterial partial pressure of oxygen to fraction of inspired oxygen (P/F) < 300 mm Hg, respiratory frequency > 30 breaths/min, or lung infiltrates > 50%.

#### Critical illness

Individuals who have respiratory failure, septic shock, and/or multiple organ dysfunction.

All the adult patients admitted in our COVID ICU from the 8th of October 2020 to the 15th of June 2021 were considered eligible if at least 1 KL-6 measurement was available at the time of their ICU admission. Terminal illness and pre-existent lung fibrosis were exclusion criteria.

### Data sources/measurement

KL-6 was measured with commercial kit produced by Tosoh (ST AIA-PACK KL6) following producer instructions [12] on left over blood samples, on the 1st and 5th day of patients ICU stay.

Numerous variables were analysed, including age, sex, body mass index, comorbidities, and severity scores (P/F ratio, Sequential Organ Failure Assessment (SOFA) score [13]).

The study sample was divided in 2 groups: group A with KL-6 serum concentration at ICU admission lower than the median KL-6 concentration and group B with KL-6 higher than the median value at ICU admission (median log-transformed KL-6 value: 6.73 U/ml). Imaging data were analysed by considering the number of interested pulmonary lobes, the presence of consolidations, crazy paving, emphysema, pneumomediastinum, and pneumothorax at CT scan.

### Variables

The primary aim of this study was to assess the association between KL-6 serum levels and clinical severity scores in adult COVID-19 patients during the second pandemic wave, in order to improve early diagnosis and defining severity of COVID-19 and to provide references for clinical and laboratory research. In addition, we investigated the association between KL-6 serum levels with SOFA score and ICU COVID-19 mortality.

### Statistical methods

The distribution of variables was evaluated by the Kolmogorov–Smirnov test and with graphical evaluation. Variable with positively skewed distribution were log transformed. At baseline, continuous variables were compared by student *T*-test or Mann–Whitney test and reported as mean ± standard deviation or median (interquartile range), according to the distribution. Categoric variable was reported as percentage, and comparison between groups was performed through the Pearson’s chi-square test. Linear regression or logistic regression analyses were performed to assess the association between KL-6 and other variables. The Kaplan–Meier analysis and Cox regression were performed in the whole sample as well as patients with KL6 below and above the median. Youden index was used to compute the value which maximizes sensitivity and specificity.

We used SPSS version 24, Chicago, IL, USA, and MedCalc.

## Results

### Participants and descriptive data

One-hundred twenty-two severe or critically ill patients, out of 150 admitted to the Salerno University Hospital COVID ICU from the 8th of October 2020 to 15th of June 2021, were included in this retrospective cohort study; baseline characteristics of source population are listed in Table 1.

**Table 1:** Baseline characteristics of source population. Data are presented as mean ± SD for continuous variables normally distributed; percentages are reported for categorical variables

Variables	Complete sample *n* = 122 Median values	Group A *n* = 61	Group B *n* = 61	*p*
Age	67 ± 11	67 ± 13	68 ± 9	0.443
Sex: M/F	31/91	15/46	16/45	0.907
Hypertension	58 (47.5%)	24 (39.3%)	34 (55.7%)	0.102
Obesity	17 (13.9%)	9 (14.8%)	8 (13.1%)	0.794
Diabetes	36 (29.5%)	20 (32.8%)	16 (26.2%)	0.552
CV pathologies	26 (21.3%)	12 (19.7%)	14 (23.0%)	0.825
Autoimmune disease	2 (1.6%)	1 (1.6%)	1 (1.6%)	1.000
Malignancies	6 (4.9%)	3 (4.9%)	3 (4.9%)	1.000
Respiratory diseases	23 (18.9%)	11 (18.0%)	12 (19.7%)	0.817
CKD	4	3	1	0.61
Length of stay (ICU days)	5 [3–9]	5 [3–9]	5 [4–8]	0.31
P/F	126 ± 59	146 ± 69	108 ± 40	0.001
SOFA	5 [3–7]	4 [3–6]	6 [4–8]	0.003
WBC day 1 10^3^/mm^3^	9.8 [7.4–13.2]	9.2 [7–11.8]	11.5 [8.1–14.3]	0.169
PCT day 1	0.15 [0.08–0.71]	0.11 [ 0.07–0.32]	0.32 [0.1–1.0]	0.088
SAPS II	28.0 ± 10.6	25.7 ± 11.3	30.8 ± 10.3	0.02
CT scan	29	16	13	
Lobes involved	5 [5–5]	5 [4, 5]	5 [5–5]	0.45
Consolidations	22 (76%)	11 (69%)	11 (85%)	0.410
Crazy paving	6 (21%)	2 (13)	4 (31%)	0.364
Cavitations	2 (7%)	0 (0%)	2 (15%)	0.192
Pneumomediastinum	7 (24%)	2 (13%)	5 (38%)	0.192
Pneumothorax	5 (17%)	1 (6%)	4 (31%)	0.14
Emphysema	6 (21)	3 (27%)	3 (38%)	1.00
P/F (PaO2/FiO2)				< 0.001
< 100 (%)	40	11	29	
100–200 (%)	72	41	31	
> 200 (%)	10	9	1	
`Respiratory support				0.003
NIV (%)	63	41	22	
IOT (%)	39	13	26	

*NIV* Noninvasive ventilation, *IOT* Orotracheal intubation

Non-survivors (*n* = 77) were older (70 ± 10 years) than survivors (63 ± 13yrs). ARDS was diagnosed at the ICU admission as severe in 49% of cases, moderate in 38%, and mild in 4%; mean P/F value at ICU admission was 125 ± 55, mean SOFA score 5.5 ± 3.0. The most frequent comorbidities were hypertension, diabetes, and cardiovascular pathologies.

### Main results

**Fig. 1:**
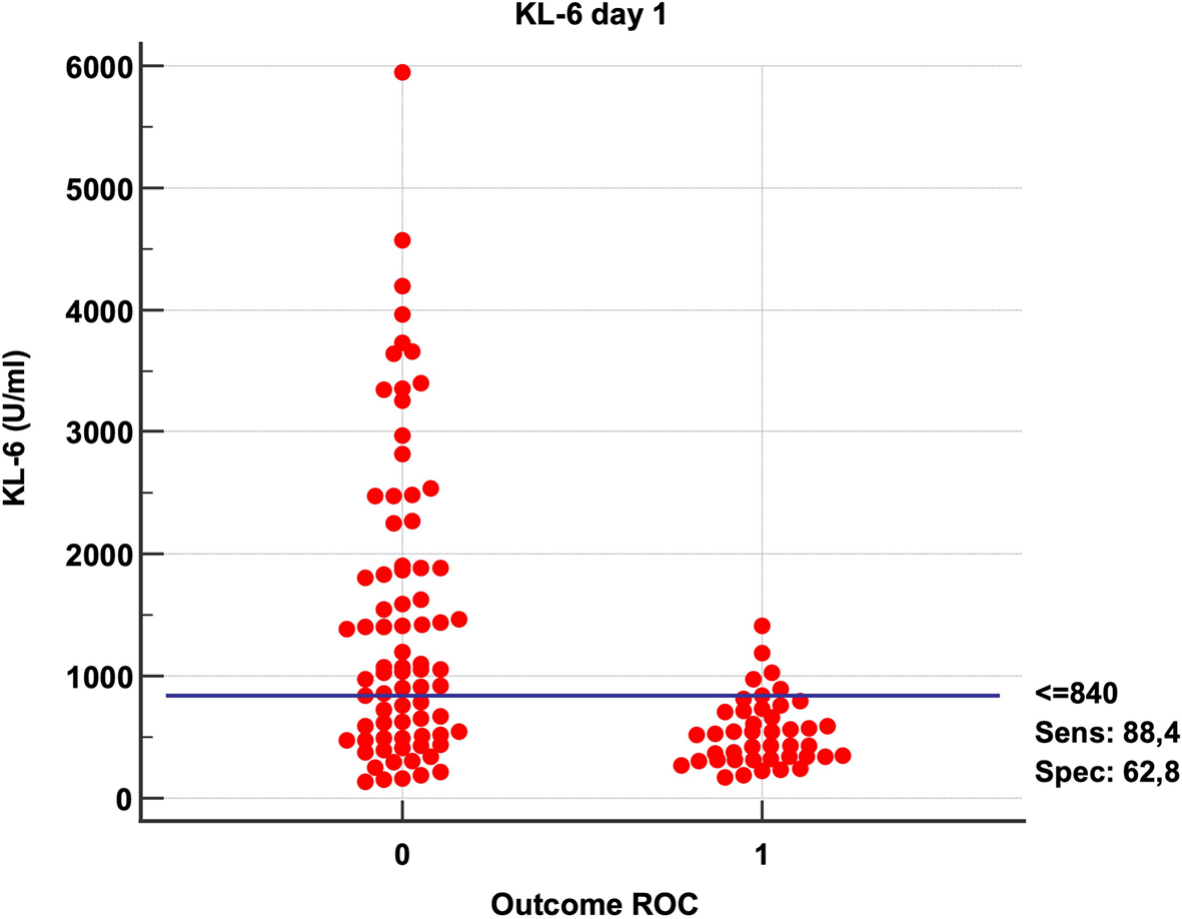
Dot plot diagram comparing KL-6 values measured on admission comparing patient who survived (column 1) with patient who did not survive (column 0). If the cutoff = 840 U/ml, KL-6 showed a sensibility of 88.4% and a specificity of 62.8%

**Fig. 2:**
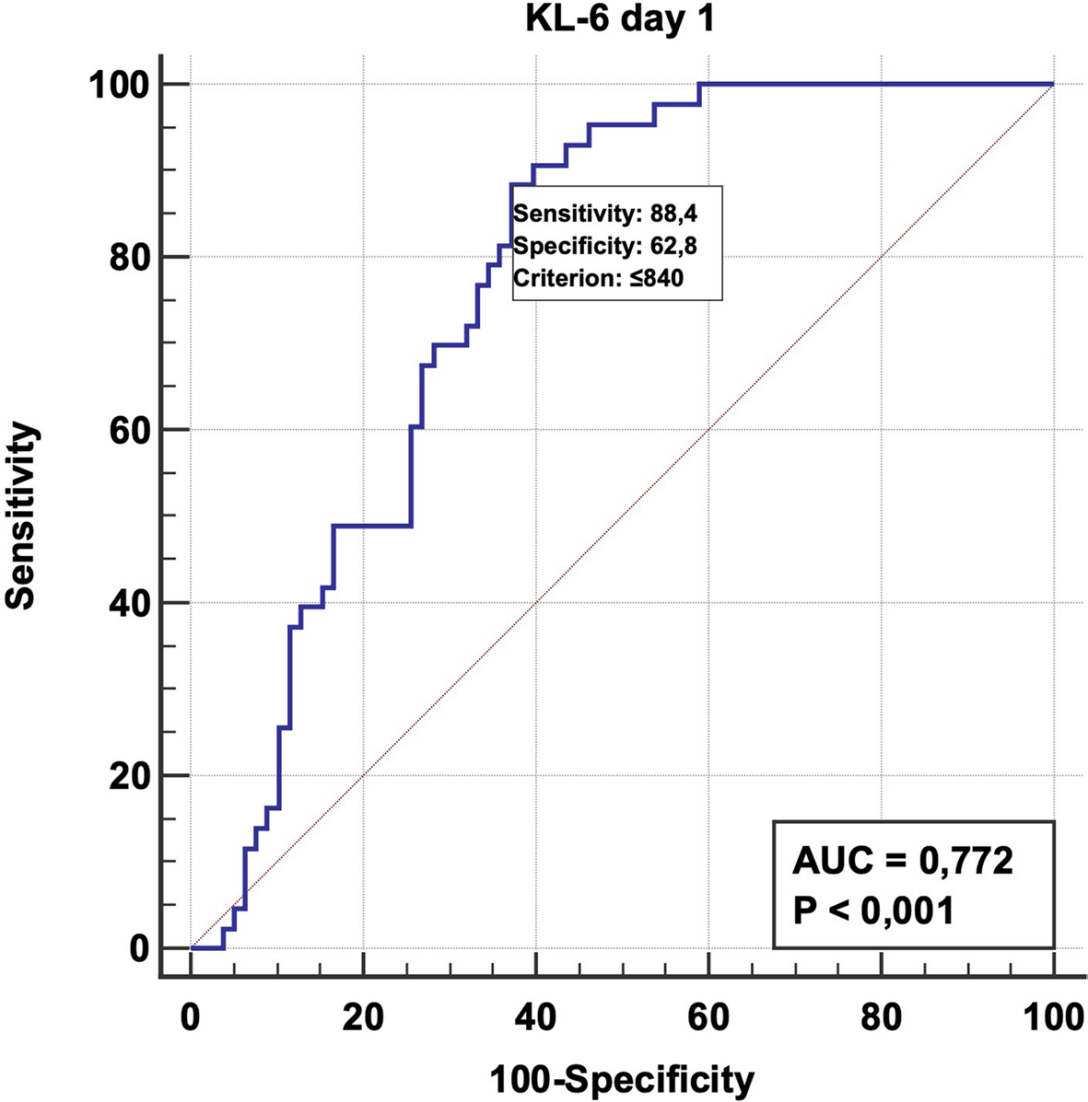
ROC curve analysis of SOFA score on the day of ICU admission. Patients with a KL-6 value above 840 U/ml may experience worse outcome (sensitivity 88.4% and a specificity of 62.8%)

At ICU admission, KL-6 serum level was significantly lower (*p* = 0.00048) in the survivor’s group (KL-6 median value 545 U/ml vs 1070 U/ml measured at admission in those patients who did not survive) (Figs. 1 and 2) and in those patients that were managed by noninvasive ventilation (NIV) for the whole length of their ICU stay (KL-6 median value 711 U/ml vs 1073 U/ml, measured in patients who required endotracheal intubation at admission or during their ICU stay) (Table 2). At 5 ICU days, the difference between survivors and patients who later would die was still significant (*p* 0.029).

**Table 2:** Baseline KL-6 at ICU admission (mean ± SD)

	Non survivors (77 pts.)	Survivors (45 pts.)	Endotracheal intubation	NIV
KL-6 U/ml	1516 ± 1236	845 ± 936	1693 ± 1305	1081 ± 1076
	*p* = 0.0004		*p* = 0.012	

For research purpose, the study sample (122 patients) was divided in two groups, according to the median log-transformed KL-6 value (6.73 U/ml): group A (61 patients with KL-6 lower than the median) and group B (KL-6 higher than the median). Differences between the two groups, regarding clinical variables as SOFA score and comorbidities, and laboratory data collected at ICU admission as procalcitonin (PCT), white blood cells count, P/F are summarized in Table 3. Group B patients were more hypoxic at admission and aggravated by more organ failures. Mortality rate was higher in group B than in group A (80.3 versus 45.9%; *p* < 0.001). We did not detect significant differences in radiological features. Instead, group A and group B differed for P/F value (*p* 0.002) and SOFA score (*p* 0.005), while they were similar for age, sex, comorbidities distribution, WBC count, and PCT values, when evaluated by univariate linear and logistic regression analysis (Table 3). Pneumothorax incidence showed a trend of significance with KL-6 values in the linear regression, but this was not confirmed in the logistic analysis. P/F was significantly and inversely related to KL-6 (*OR* 0.99; 95% *CI*: 0.978/0.998; *p* 0.009) (Table 4).

**Table 3:** Univariate linear and logistic regression analysis. The two groups differed for P/F value (*p* = 0.003) and SOFA (*p* = 0.005)

Variables	Linear regression			Logistic regression		
	*β*	95% *CI*	*p*	Odds ratio	95% *CI*	*p*
Age	0.001	− 0.01/0.02	0.916	1.01	0.98/1.05	0.424
Sex: M/F	0.101	− 0.255/0.576	0.576	0.92	0.41/2.07	0.917
Hypertension	0.206	− 0.103/0.514	0.190	1.94	0.94/3.99	0.071
Obesity	0.033	− 0.415/0.481	0.885	0.87	0.31/2.43	0.872
Diabetes	− 0.002	− 0.342/0.339	0.992	0.73	0.33/1.59	0.428
CV pathologies	0.183	− 0.194/0.561	0.338	1.21	0.51/2.90	0.659
Respiratory pathologies	0.137	− 0.259/0.533	0.495	1.11	0.45/2.76	0.817
Autoimmune disease	− 0.165	− 1.38/1.057	0.789	1.00	0.06/16.4	1.00
Malignancies	− 0.083	− 0.801/0.635	0.819	1.00	0.194/5.161	1.00
P/F	− 0.004	− 0.006/ − 0.001	0.007	0.99	0.979/0.995	**0.002**
SOFA	0.071	0.021/0.122	0.006	1.21	1.06/1.39	**0.005**
WBC day 1 10^3^/mm^3^	0.026	0.001/0.05	0.038	1.06	0.99/1.13	0.065
PCT ng/ml	0.00023	− 0.010/0.011	0.925	1.012	0.98/1.05	0.483

**Table 4:** In both linear and logistic multivariate analyses, P/F was significantly and inversely related to KL-6

Variables	Linear regression			Logistic regression		
	*β*	95% *CI*	*p*	*OR*	95% *CI*	*p*
Age	− 0.003	− 0.018/0.012	0.695	1.004	0.96/1.04	0.834
Sex: M/F	0.153	− 0.218/0.525	0.415	1.18	0.46/3.03	0.740
P/F	− 0.003	− 0.006/ − 0.0005	0.023	0.99	0.978/0.998	0.009
SOFA	0.041	− 0.016/0.098	0.158	1.14	0.97/1.32	0.114
WBC day 1 10^3^/mm^3^	0.020	0.004/0.044	0.107	1.04	0.97/1.12	0.250

### Outcome data

**Fig. 3:**
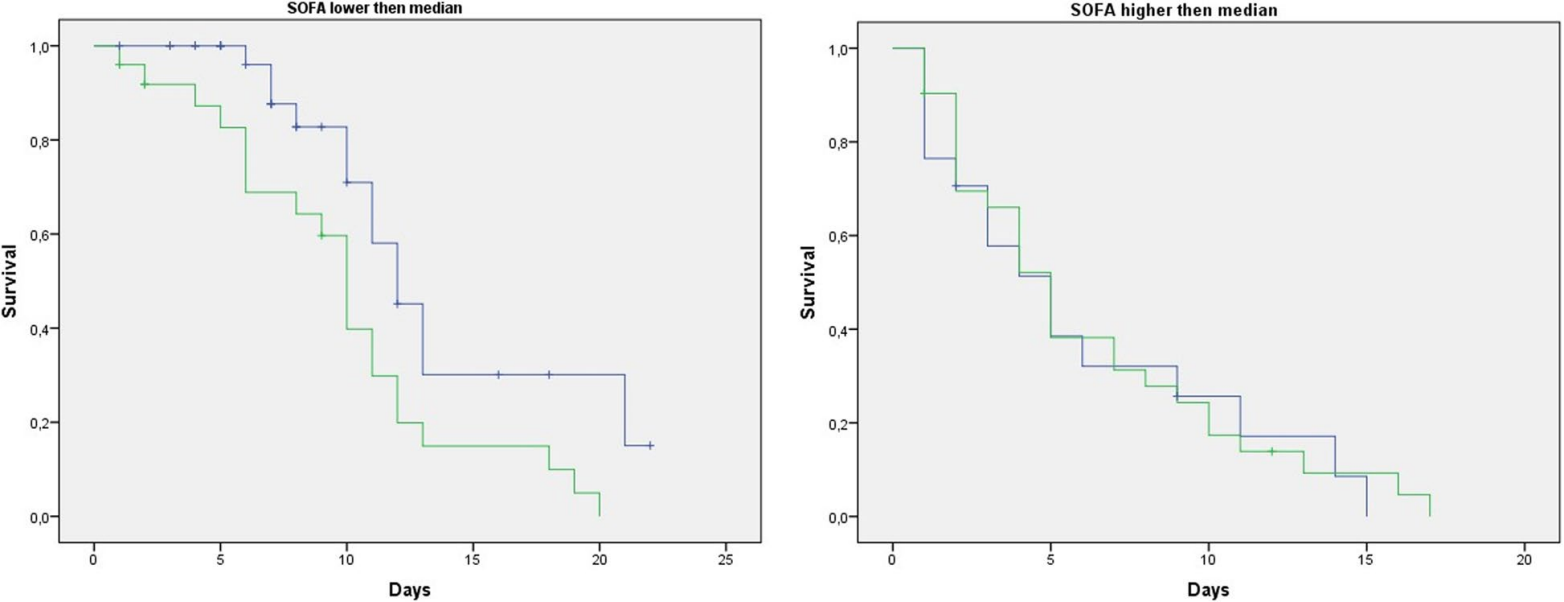
Kaplan–Meier curves, dashed blue line represents group A; the solid green line represents group B. Upper panel, A and B group patients with SOFA value at admission lower than median SOFA; lower panel, A and B group patients with SOFA value at admission higher than median (SOFA median value = 5). Upper panel, higher cumulative probability of survival on group A compared to group B when SOFA was < 5. Lower panel, similar cumulative probability of survival in both groups (median 6 vs 5 days in group A and group B, respectively). KL-6 was a solid prognostic marker when SOFA lower than 5, while when SOFA was higher than 5, in the most severe patients, its prognostic power was not statistically significant

To understand the prognostic role of KL-6 in the subgroup of patients who were already sicker at ICU admission (as shown by their SOFA score, higher than the median value of SOFA calculated in the whole population, which was 5), a Kaplan–Meier analysis was performed. It showed a significant higher mortality in patients who had KL-6 value at admission > 6.73 U/ml and SOFA score < 5.0 (median value of SOFA in this population), while in the subgroup with *SOFA* > 5.0 (higher than the median SOFA), the impact of KL-6 on mortality was not evident (Fig. 3). Accordingly, the Cox regression analysis showed a significant prognostic role of KL-6 on mortality in the whole sample (*HR*: 1.78, 95% *CI* 1.12–2.82, *p* = 0.02) as well as in the subgroup with SOFA lower than the corresponding median value (*HR*: 1.86, 95% *CI* 1.02–3.44, *p* = 0.045), but not in the subgroup of patients with SOFA above the median (*HR* 0.96, 95% *CI* 0.69–1.34, *p* = 0.96). Therefore, SOFA acted as a significant effect modifier of the KL-6/mortality link (*p* = 0.048).

## Discussion

### Key results

In this retrospective prognostic study, we correlated KL-6 values at ICU admission with disease severity in COVID-19 patients during the second wave of the pandemic in a COVID-dedicated hospital. Our findings showed that elevated KL-6 (> 680 U/ml) was strongly associated with mortality in ICU, thereby contributing to the growing body of evidence for the utility of KL-6 in the context of COVID-19 infection.

### Interpretation

KL-6 glycoprotein is mainly expressed on alveolar type 2 cells in the lung, and it is produced more prominently by proliferating, or regenerating, injured type 2 cells than by healthy type 2 cells. The presence of KL-6 has been used to monitor severity of disease in idiopathic pulmonary fibrosis [8]. Liu et al. [14], in a lung proteomics animal experimental research (rhesus monkeys), quantified the overall difference of protein expression pattern between control and COVID-infected groups. When compared to the control group, in the lung, 757 proteins were differentially expressed in the infected group. Our study did not check total protein expression in the lungs of COVID patients, but it aimed to identify an easy and available biomarker that physicians could use to risk stratify COVID-19 patients. The use of biomarkers to predict disease severity has proven essential for resource allocation, particularly for respiratory support needs. In a previous study on a population of 67 COVID survivors [15], median KL-6 was 365 U/ml (IQR 233–493), and the authors concluded that “high KL-6 levels at 12 weeks with persisting CT abnormalities (GGO/fibrosis) is a finding that requires further exploration”. D’Alessandro et al. [16], in 14 severe COVID patients, found very elevated serum KL-6 concentrations (median *IQR*, 1125; 495–2034). These previous works, with the others included in the above-cited meta-analysis [10], let the search for cutoff values in COVID patients still open. KL-6 pathophysiological role in lung diseases is not completely understood. KL-6 belongs to the mucin family, and proteins aimed to improve mucosal barrier integrity and functionality. Hurasawa et al. identified KL-6 as human MUC1 [17]. Mucins, both secreted or cell bound, create a protective mucus layer on the host cells, reducing pathogens access to their receptors. Up to now, MUC1/KL-6 has been studied as a diagnostic marker for respiratory disease severity, but there has been no extensive research assessing the mechanism of its overexpression. Whether a mechanistic proof is to be done (does KL-6 cause some aspect of COVID pathophysiology?) or if a prediction model only is possible (KL-6 adds to other markers to discriminate an outcome, such as mortality) should be clarified. Kost-Alimova et al. [18] identified fostamatinib, which was previously approved by the FDA for chronic immune thrombocytopenia, as a drug that significantly reduces MUC1 protein abundance. Their experimental findings indicated the potential of repurposing fostamatinib for the treatment of CARDS (https://clinicaltrials.gov/ct2/show/NCT04579393), even if, unfortunately, KL6/MUC1 has already been a failed target in oncology [19].

Lorenzoni et al. [20], through a machine learning approach for predicting ICU mortality in COVID-19 patients, proved that age was the leading predictor, followed by total SOFA score at ICU admission, and the P/F used for SOFA calculation. In our previous experience [21] and in the present study too, patients’ characteristics at ICU admission severely conditionate the success of advanced therapeutic strategies. In this paper, when SOFA score is higher than 5, prognosis is already compromised, and consequently, the prognostic power of KL-6 is reduced, while it can still be helpful to identify lung injury in patients with a lower organ failure score (*SOFA* < 5).

Ru et al. [22] reported that mean serum PCT levels were over eight times higher in critical COVID patients than in moderate patients. PCT levels may be associated with bacterial coinfection which might justify such an increase. In our experience, PCT was not a marker of severity of COVID lung failure at ICU admission. Nevertheless, we think that an increase in PCT should always be investigated for the presence of a concomitant bacterial infection.

Changes in KL-6 serum levels may be related to barotrauma and volutrauma too, even if it is very difficult to distinguish the origin of the biomarker release in a complex context as COVID ARDS.

### Limitations

The main limitation of this research is that is an observational study with retrospective enrolment. Moreover, we think that KL-6 value as prognostic index should be tested in different COVID populations or timing, i.e. at emergency room admission, and the dynamic of its changes should be tested, to understand if it shows prognostic value for long-term COVID effects on the lung functions. In our retrospective study, in fact, multiple unmeasured variables may have affected the outcomes. Conclusions should be validated by larger, definite prospective studies in the future.

## Conclusions

This paper supports the possibility to use KL-6 in a panel of biomarkers, which may help clinicians to stratify mortality risk and the need for ICU admission. It would be very advantageous to ascertain firm cutoff values for this purpose.

## Data Availability

The datasets used and/or analysed during the current study are available from the corresponding author on reasonable request.
